# *Karenia brevis* Extract Induces Cellular Entry through Distinct Mechanisms in Phagocytic RAW 264.7 Macrophages versus Non-Phagocytic Vero Cells

**DOI:** 10.3390/md22010004

**Published:** 2023-12-19

**Authors:** Laurie A. Minns, Kathryn T. Sausman, Ariel P. Brown, Robert A. York, Jennifer R. McCall

**Affiliations:** 1 School of Nursing, College of Health and Human Services, University of North Carolina Wilmington, 601 S. College Road, Wilmington, NC 28403, USA; minnsl@uncw.edu (L.A.M.);; 2 Center for Marine Science, University of North Carolina Wilmington, 5600 Marvin K Moss Lane, Wilmington, NC 28409, USA; 3 Algal Resources Collection, University of North Carolina Wilmington, 5600 Marvin K Moss Lane, Wilmington, NC 28409, USA

**Keywords:** flow cytometry, autofluorescence, immunofluorescence, macrophages, Vero cells

## Abstract

Marine algae extracts are an important area of potential drug discovery; however, nearly all studies to date have used non-fluorescent-based methods to determine changes in target cell activity. Many of the most robust immunological and cellular analyses rely on fluorescent probes and readouts, which can be problematic when the algae extract is fluorescent itself. In this study, we identified the fluorescent spectrum of an isolated extract from the marine dinoflagellate *Karenia brevis*, which included two fluorescing components: chlorophyll α and pheophytin α. When excited at 405 nm and 664 nm, the extract emitted fluorescence at 676 nm and 696 nm, respectively. The extract and its fluorescing components, chlorophyll α and pheophytin α, entered phagocytic RAW 264.7 macrophages and non-phagocytic Vero kidney cells through distinct mechanisms. When incubated with the extract and its main components, both the RAW 264.7 macrophages and the Vero cells accumulated fluorescence as early as 30 min and continued through 48 h. Vero kidney cells accumulated the *K. brevis* fluorescent extract through a dynamin-independent and acidified endosomal-dependent mechanism. RAW 264.7 macrophages accumulated fluorescent extract through a dynamin-independent, acidified endosomal-independent mechanism, which supports accumulation through phagocytosis. Furthermore, RAW 264.7 macrophages downregulated cell-surface expression of CD206 in response to extract stimulation indicating activation of phagocytic responses and potential immunosuppression of these immune cells. This study represents the first characterization of the cellular update of *K. brevis* extracts in phagocytic versus non-phagocytic cells. The data suggest the importance of understanding cellular uptake of fluorescing algae extracts and their mechanism of action for future drug discovery efforts.

## 1. Introduction

*Karenia brevis*, a dinoflagellate first identified due to its production of brevetoxin, a toxin that causes harm to humans and marine organisms, is a complex organism containing extracts that may also provide benefit as potential new therapeutic targets [1,2,3]. *K. brevis* grows under multiple light conditions and fluoresces at a variety of wavelengths due to environmental influences and the activity of its photosystem II [4]. Natural fluorescence emission associated with *K. brevis* blooms ranges from 340 nm to 495 nm and can be useful in detection of this harmful algae [5]. For example, increases in chlorophyll α is one of several indicators of *K. brevis* environmental blooms [5,6]. Most of the work on *K. brevis* has focused on its harmful toxic effects in both human and marine species [7,8]; however, this alga also demonstrates therapeutic potential as evidenced by beneficial effects of *K. brevis*-derived natural products in stroke recovery, asthma, inflammation, and other disease models [9,10,11,12]. An important component missing from these studies is a precise mechanistic understanding of how *K. brevis* extracts, other than brevetoxins, interact directly with target cells. In fact, new studies have come to light showing that canonical mechanisms of brevetoxin interaction with sodium channels may not be the only pathway of cellular effect [13,14,15,16].

Extracts from several other species of algae have anti-tumor, anti-obesity, anti-diabetic, and anti-inflammatory properties, yet these reports do not identify how the algae components interact directly with cells themselves [17,18,19,20,21]. It is possible characterization of cellular uptake is due to the fluorescent properties of the extracts themselves. For example, water extracts from *Palmaria palmata*, a red alga found in high latitude zones of the Atlantic and Pacific oceans, contain components with absorption peaks at 320 nm, 495 nm, 565 nm, and 610 nm, absorbances that could interfere with fluorescent-based assays [22]. To date, experiments on the biological activity of most fluorescing algae extracts have largely focused on discrete cellular endpoints (e.g., cytokine secretion), which never exist in a vacuum in vivo and fail to consider drug effects on signaling pathways in cells. Enzymatically digested extracts from *P. palmata* suppress inflammatory responses in RAW 264.7 macrophages by decreasing iNOS, TNF-α, and IL-6, yet it is largely unknown how cellular uptake of its fluorescent extract influences immune responses [22]. To date, the mechanisms by which fluorescing algae extracts enter host cells are not well characterized.

Cell-based assays, particularly those with fluorescent components such as flow cytometric analysis and fluorescent microscopy, are useful for illuminating the complex spectrum of drug effects on target cells. These assays can elucidate impacts on inflammation signaling pathways, as well as detect potential toxic or off-target side effects on individual cells. Such tools allow for reduced risk of many of the common failures of clinical trials [23]. Flow cytometry is a powerful tool in drug discovery because it allows for multiparameter analysis of a given treatment on individual cells stained with antibodies conjugated to fluorochromes for specific antigen detection and quantification [24,25]. Flow cytometry also allows for targeted analysis of different cellular populations, which can increase the power of the experiment [26]. In addition to flow cytometry, fluorescence cellular imaging can shed light on how cells change when stimulated by novel natural agents and drugs. When the compound of interest is fluorescent on its own, it provides the opportunity to determine how it interacts with host cells directly by quantifying and characterizing intracellular fluorescence. 

Natural product drug discovery requires bioassay testing to determine biological effects of a potential therapeutic. Pan-assay interference compounds (PAINS) can slow the progression of drug discovery due to a variety of false positives in bioassays [27]. Regardless, several FDA-approved small molecule drugs contain known PAINS motifs and display bioactivity; therefore, excluding molecules due solely to the presence of PAINS motifs could hinder drug discovery [28]. Although there are several known compounds containing PAINS motifs, fluorescing components such as chlorophyll and related chemical compounds have yet to be considered as PAINS or PAINS-like compounds. In depth and detailed pre-clinical experimentation can help reduce the cost of the clinical trial process, which is estimated to cost close to a billion dollars and approximately ten years to bring a new drug to market [29,30]. 

When considering how a novel therapeutic agent can influence cellular responses, the cell type assayed must also be carefully considered. Vero cells (derived from the African green monkey kidney) are widely accepted as being non-phagocytic cells, and, therefore, offer an appropriate control to determine whether target cells accumulate fluorescence when incubated with chlorophyll-containing marine algae extracts [31,32]. RAW 264.7 macrophages are appropriate to determine early immune responses and have phagocytic activity. Macrophages are useful in early drug discovery since they are immune sentinel cells, initiators of the immune response, and polarize to M1 or M2 phenotypes to direct downstream inflammatory responses [33,34,35]. Macrophages play an essential role in the tumor microenvironment, liver disease, autoimmunity, and many more disease states [34,36,37,38,39]. Macrophages are also phagocytic cells, responsible for engulfing pathogens and cellular debris during infections and inflammation. The purpose of this study was to determine how phagocytic cells such as macrophages interact with fluorescing marine algae extract from *Karenia brevis* compared to non-phagocytic cells (Vero cells) using fluorescent-based cellular assays.

## 2. Results

### 2.1. K. brevis Algal Extracts Absorb and Emit Fluorescence 

**Figure 1:**
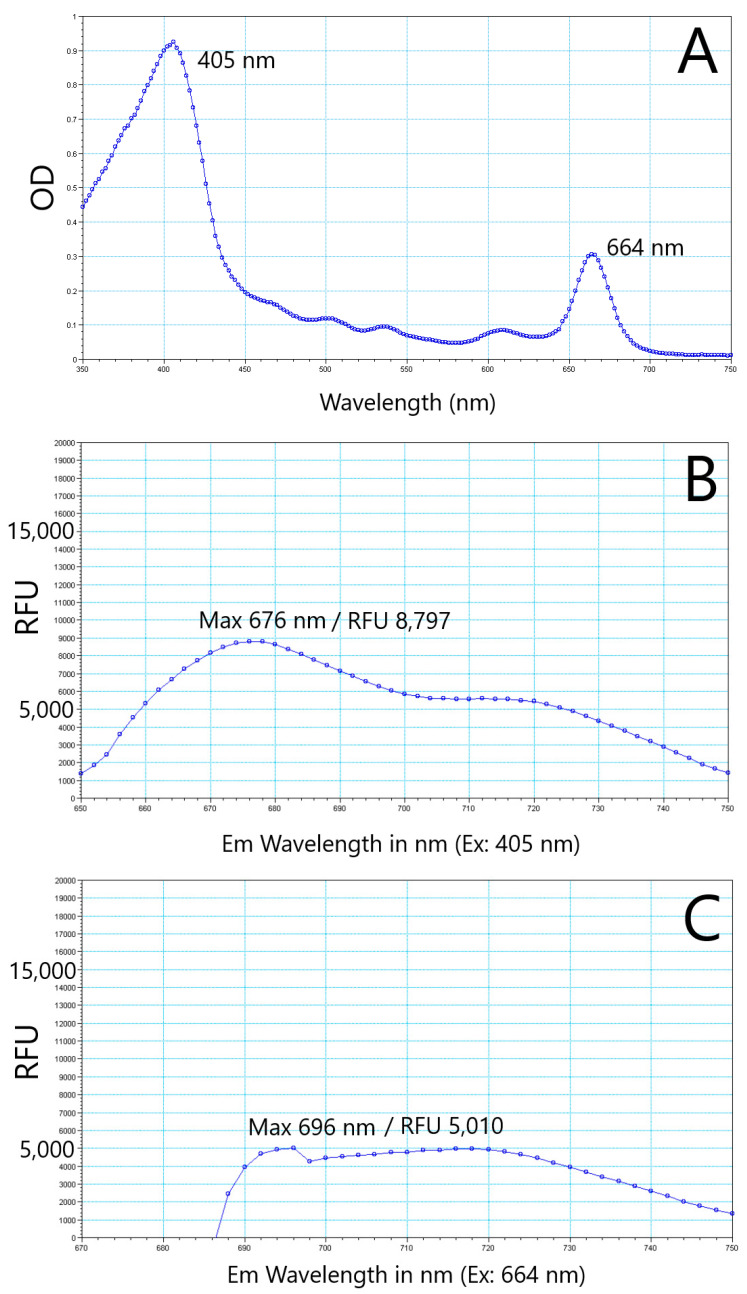
*K. brevis* algal extracts absorb and emit fluorescence at multiple wavelengths. Spectral properties of *K. brevis* extract at 1 mg/mL. Extracts absorbed light with peak wavelengths at 405 nm and 664 nm (panel (**A**)). Extracts were then excited with peak wavelengths (405 nm: panel (**B**) or 664 nm: panel (**C**)).

Briefly, *K. brevis* cultures were extracted using liquid-liquid extraction, homogenized, and then partitioned using liquid-liquid extraction again. The petroleum ether fraction of the liquid-liquid extraction was partitioned using flash chromatography. One of the peaks was collected and tested at multiple concentrations using a FlexStation 3, a fluorescence plate reader. An absorption scan was performed at 1 mg/mL extract concentration, resulting in two peaks at 405 nm and 664 nm (Figure 1A). When excited at 405 nm and 664 nm, the extract emitted fluorescence with maximum peaks at 676 nm and 696 nm, respectively (Figure 1B,C). Due to excitation wavelength interference in the red range, peak fluorescence from 664 excitation was more difficult to discern precisely (Figure 1C). Fluorescence interference occurs due to insufficient spectral separation from the excitation light and detection of the emission wavelength on the FlexStation 3 [40]. To further confirm the excitation, the extract was also excited at the blue and red wavelengths used by flow cytometer lasers (488 nm and 640 nm), which is important for later characterization assays. Peak fluorescence from 488 nm excitation was 680 nm (not shown) but was approximately 75% less intense (as measured by RFUs) than excitation at the peak absorbance value of 405 nm (Figure 1). Peak fluorescence from 640 nm excitation was 694 nm, but again, fluorescence interference from bleed over in the excitation wavelength contributed to irregular response curves. 

### 2.2. Fluorescent K. brevis Extracts Contain Both Chlorophyll α and Pheophytin α

**Figure 2:**
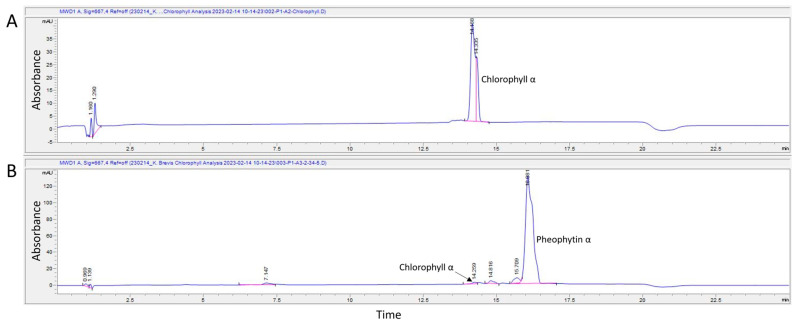
HPLC-UV chromatogram with detection wavelength at 667 nm of a chlorophyll α reference standard (panel (**A**)) and *K. brevis* algae extract (panel (**B**)). The chromatogram for the algae extract indicates that although chlorophyll α was present, pheophytin α was most abundant. Mass spectroscopy later identified Pheophytin α as the large peak in panel (**B**).

Algae are photosynthetic organisms that contain fluorescent pigments such as chlorophylls, pheophytins, and carotenoids. Since chlorophyll α is the most abundant pigment in all microalgae [41], it was expected to be present in the *K. brevis* extract. The extract was dark green in color when dissolved in methanol and was analyzed against a chlorophyll α reference (provided by the Aquatic Ecology Laboratory Research Team at the University of North Carolina Wilmington) on HPLC with a detection wavelength of 667 nm to determine if chlorophyll α was the dominant compound in the extract. Based on the analysis, chlorophyll α was present in the extract but was not the dominant compound (Figure 2). Retention time of chlorophyll α was 14.2 min, but another peak at 16.1 min had a higher peak area, indicating a higher concentration of this second compound.

From the HPLC data, the peak with the largest area was isolated and separated from chlorophyll α. When the isolated compound was dried and resolubilized in methanol, the color of the solution was grayish green. When analyzed by mass spectrometry, the spectrum indicated that the compound with the highest concentration had a molecular mass + hydrogen ion (M + 1) of 873 Da (Figure S1). This M + 1 suggests that pheophytin α, a derivative of chlorophyll α formed in acidic conditions when the magnesium atom is replaced with two hydrogens [42], is likely present in the isolated algal extract. An M + 1 of 895 Da was also detected. This M + 1 value could correspond with residual chlorophyll α, but it is unlikely since chlorophyll α had been removed from the extract in the previous HPLC purification. It is more likely to correspond with the presence of a sodium adduct, which is commonly detected in positive mode. Based on the mass spectrum and color of the solution isolated from the original algal extract, the isolated compound is consistent with pheophytin α; pheophytin α has peak absorption maximums around 410 and 665 nm and fluoresces around 670 nm [43,44].

### 2.3. Assessment Fluorescence in Phagocytic Macrophages and Non-Phagocytic Vero Cells Using Flow Cytometry

**Figure 3:**
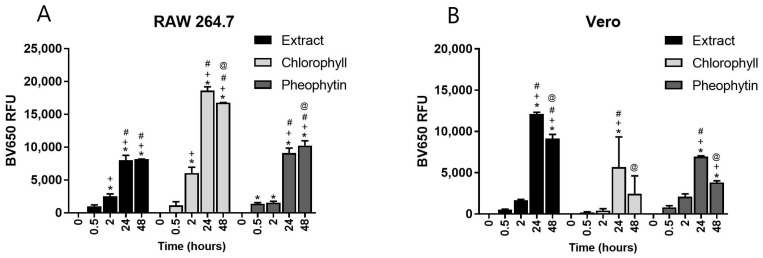
Fluorescence accumulation in RAW 264.7 cells (Panel (**A**)) and Vero cells (Panel (**B**)) over a 30 min to 48 h time course as excited by violet laser and read on Brilliant Violet 650 filter. Symbols indicate a statistically significant difference from no treatment (*), 0.5 h (+), 2 h (#), and 24 h (@) with *p* < 0.05.

**Figure 4:**
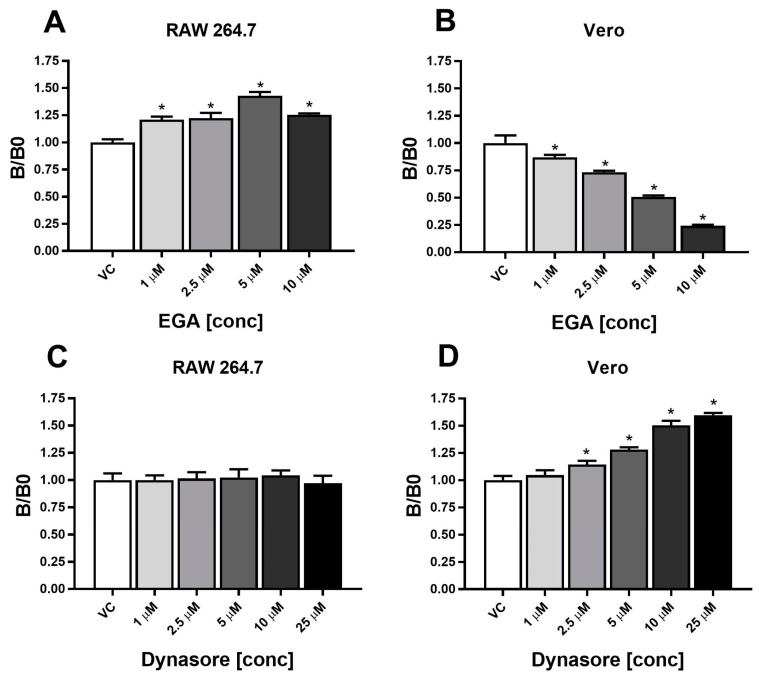
RAW 264.7 macrophages accumulate more *K. brevis* extract fluorescence in the presence of EGA, a mem-brane trafficking inhibitor (Panel (**A**)), whereas Vero cells display a decrease in fluorescence in a dose-dependent manner with EGA pre-treatment (Panel (**B**)). RAW 264.7 showed no change in fluorescence accumulation in the presence of Dynasore, a dynamin-dependent inhibitor (Panel (**C**)). Non-phagocytotic Vero cells show an increase in extract fluores-cence uptake in a dose-dependent manner when treated with Dynasore (Panel (**D**)). * *p* < 0.05 when compared to vehicle control.

To determine how the *K. brevis* extract, chlorophyll α, and pheophytin α interact with RAW 264.7 macrophages and non-phagocytic Vero cells, cells were incubated with each component at 500 ng/mL over a time course of 30 min, 2 h, 24 h and 48 h. A non-toxic dose of 500 ng/mL was decided for cellular assays. Cells were washed, and fluorescence accumulation was measured via flow cytometry using the emission filter for Brilliant Violet, 650 nm. RAW 264.7 macrophages began accumulating fluorescent extract within 30 min, as detected by the violet laser (excited by the 405 violet laser and emitting at 650 nm) (Figure 3A) and the red laser (excitation at 640 nm and emission at 671 nm) (Figure S2). Surprisingly, the non-phagocytic Vero cells also began accumulating fluorescent extract within 30 min and peaked at 24 h when incubated with each of the individual components (Figure 3B). Notably, the chlorophyll α accumulated more in the RAW 264.7 cells as indicated by an increase in RFUs over the extract or pheophytin α (Figure 4A). Data from these experiments demonstrate fluorescence from the extract and its primary components accumulate in both phagocytic and non-phagocytic cells. Importantly, when RAW 264.7 cells were treated with *K. brevis* extract for 24 h and assayed by flow cytometry with all filters available, the natural fluorescence interfered with over half of the flow cytometer filters due to the excitation and emission wavelengths of the *K. brevis* extract itself (Table S1). 

### 2.4. Fluorescence Accumulates through Distinct Mechanisms in RAW 264.7 and Vero Cells

Mammalian cells endocytose materials through a variety of mechanisms independent of phagocytosis, including macropinocytosis, clathrin-mediated endocytosis, dynamin-dependent endocytosis, dynamin-independent endocytosis, and glycolipid rafts (for review, [45]). Although macrophages are well-known phagocytic cells, they can also accumulate particles through pinocytosis and non-phagocytic endocytosis depending on particle size and charge [46,47,48,49]. Vero kidney cells are non-phagocytic cells [31]. Since we observed an increase in fluorescence over time after exposure to the *K. brevis* extract and its main fluorescing components in both phagocytic and non-phagocytic cells, we sought to elucidate the mechanism of cellular entry into both cell types using inhibitors of cellular uptake. 2-[(4-bromophenyl)methylene]-N-(2,6-dimethylphenyl)-hydrazinecarboxamide (EGA) prevents acidified endosomes from entering cells [50]. Dynasore, inhibits dynamin, responsible for clathrin-coated vesicle formation [51].

**Figure 5:**
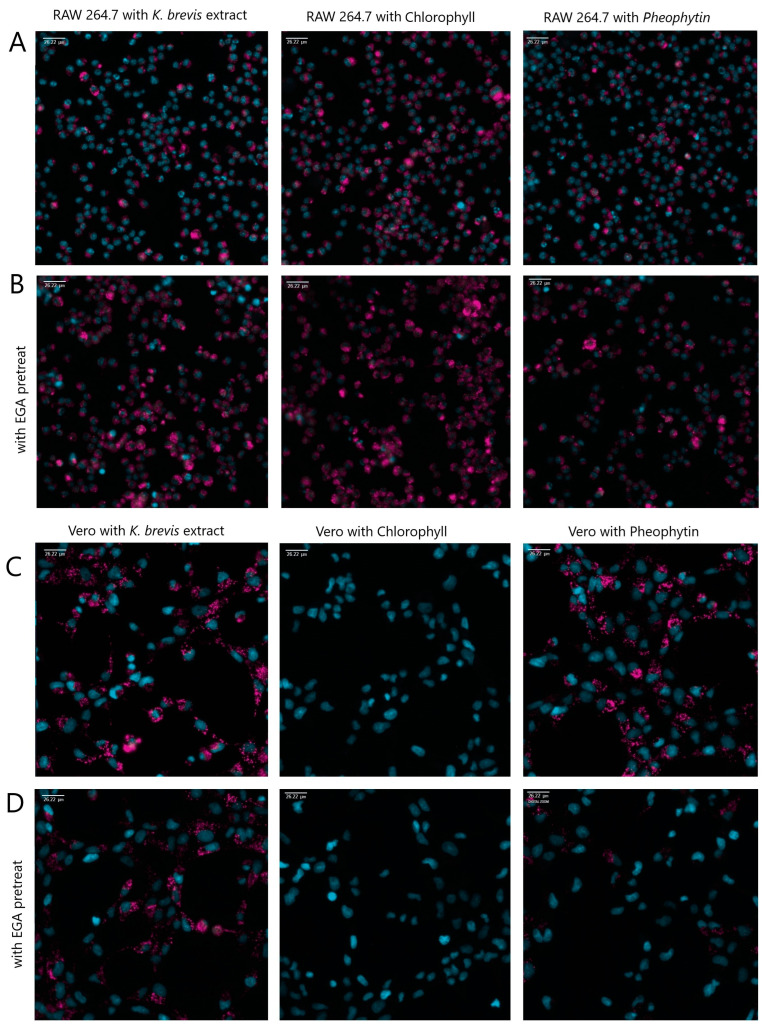
RAW 264.7 (Panel (**A**,**B**)) and Vero cells (Panel (**C**,**D**)) were pretreated with EGA for one hour (Panel (**B**,**D**)) or not (Panel (**A**,**C**)), then treated with fluorescent components of *K. brevis*, the extract, chlorophyll α, and pheophytin α (Cy5 pink color) for approximately 24 h. Cells were then treated with nucleus Hoechst stain (DAPI blue color) for 1 h, washed, and imaged using Image Xpress PICO at 20×.

To determine whether RAW 264.7 macrophages and Vero kidney cells accumulate fluorescent extract through an acidified endocytic mechanism, both RAW 264.7 cells and Vero cells were pretreated for one hour using an increasing dose response of 1 µM to 10 µM EGA prior to treatment with 500 ng/mL extract for 24 h. Fluorescence was measured using flow cytometry (Figure 4A,B). We observed a dose-dependent decrease in fluorescence in Vero cells (Figure 4B), whereas the RAW 264.7 cells continued to accumulate fluorescent extract until we reached the maximum non-toxic dose (10 μM) of EGA (Figure 4A). To confirm this observation, cells were pre-treated with 5 μM of EGA and cells were imaged using fluorescent microscopy. As with the time course fluorescence from flow cytometry (Figure 3), RAW 264.7 cells visibly accumulated chlorophyll α the most, whereas Vero cells accumulated very little visible fluorescence with chlorophyll α but showed fluorescence with the extract itself and the pheophytin α (Figure 5). Together, these data suggest that RAW 264.7 cells do not require acidified endosomes for cellular entry whereas Vero cells do. 

To determine the role of dynamin-dependent endocytosis in both the RAW 264.7 cells and the Vero cells, we pre-treated cells with Dynasore in an increasing dose response, 1 µM to 25 µM for one hour, followed with 500 ng/mL extract treatment for 24 h, and fluorescent extract accumulation was observed through flow cytometry (Figure 4C,D). Interestingly, fluorescent extract accumulation was significantly higher in the Vero cells with Dynasore pre-treatment beginning at the 2.5 μM dose whereas Dynasore did not affect fluorescent extract accumulation in the RAW 264.7 cells (Figure 4C,D). Together, these data indicate fluorescent extract in RAW 264.7 cells occurs through a dynamin-independent and acidified-endosome independent mechanism. 

### 2.5. K. brevis Extract Downregulated RAW 264.7 Expression of CD206, in the Absence of Changes in Cytokine Secretion 

**Figure 6:**
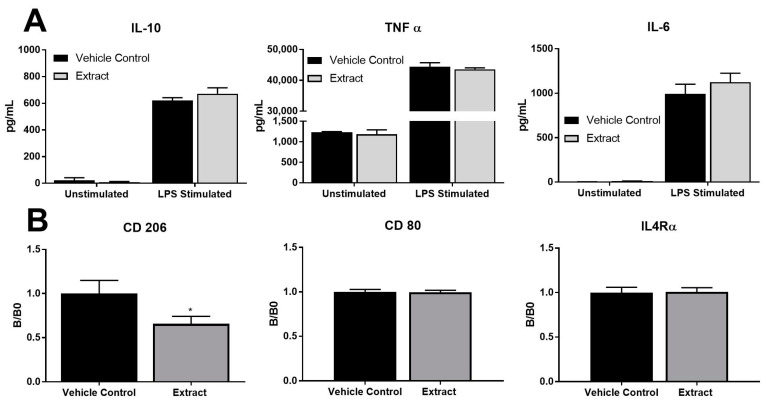
Assessment of macrophage alternative activation states by cytokine secretion ((**A**) n-3) IL-10, TNF α and IL-6 and cell surface expression (**B**) of CD206 (n-10), CD80 (n-3) and IL4Rα (n-3). RAW 264.7 macrophages were treated with algal extracts for 24 h and stained with fluorescent antibodies prior to analysis on a flow cytometer. * indicates a statistically significant difference from vehicle control (VC) with *p* < 0.05.

To determine whether our K. brevis extract was able to influence the cytokine response in activated RAW 264.7 macrophages, unstimulated and LPS-stimulated macrophages were treated with 500 ng/mL of extract for 24 h. As expected, RAW 264.7 macrophages stimulated with LPS modestly increased IL-10, TNFα, and IL-6 secretion (Figure 6A), all indicative of macrophage activation [52]. When treated with the extract, these LPS-stimulated macrophages maintained the same activity as indicated by similar levels of IL-10, TNFa, and IL-6 secretion (Figure 6A). In general, this finding is not surprising, as it is well known that LPS activates cytokine secretion from macrophages, and our data are consistent with other work that shows LPS stimulation of RAW 264.7 cells results in increased IL-10 and TNFα expression [53,54]. However, exposure of cells to the extract did not change cytokine responses due to LPS activation. Other studies have found effects on LPS-induced cytokine secretion due to extract treatment (e.g., from Carpomitra costata or polysaccharides from Sargassum horneri), but these extracts nearly certainly have different components than ours; thus, these results are not necessarily surprising [55,56]. 

M2 macrophages are characterized by high CD206 expression and IL-4Rα+, whereas M1 cells are characterized by high CD80 expression [57,58,59]. CD206 is also a mannose receptor on macrophages and is important in the phagocytosis of pathogens and regulation of inflammatory glycoproteins; it moves between the plasma membrane and the endocytic compartment [60,61]. To determine how *K. brevis* algal extracts influenced the expression of these extracellular markers, we incubated unstimulated RAW 264.7 macrophages and measured CD206, CD80, and IL4Rα cell-surface expression via flow cytometry using filters and conjugated fluorophores with a wavelength that does not detect the fluorescence of the extract itself (AF488, PE-CF594 and BV421, respectively). Low expression of CD206 is associated with an M1 macrophage, whereas a high upregulation of CD206 indicates an M2 phenotype [62]. The M2 phenotype promotes tissue self-repair by downregulating the pro-inflammatory response [63,64]. The results from this experiment showed a slight but significant decrease in CD206 of 34% (Figure 6). Together, these data suggest the RAW 264.7 cells phagocytose the extract which affects cell surface expression of CD206. The decrease in CD206 could indicate phagocytic activity or a change in polarization away from an M2 phenotype. It is important to note that decreases in cell-surface expression of CD206 did not result in a corresponding change in cytokine secretion (e.g., IL-10) from LPS-stimulated macrophages; however, these two effects are not always correlated. In fact, this finding further highlights why diverse bioassay testing is important to examine the multi-faceted effects that extracts may have on immune cells, as effects on CD206 expression may be missed if investigators relied only on cytokine secretion as a read-out.

## 3. Discussion

In our study, we sought to determine how phagocytic cells such as RAW 264.7 macrophages interact with fluorescing marine algal extract from *Karenia brevis* compared to non-phagocytic Vero cells using fluorescent-based cellular assays. We assayed the response to *K. brevis* extracts and its fluorescing components chlorophyll α and pheophytin α. Chlorophyll α is the most abundant pigment in all microalgae [42]; thus, it was expected to be present in the algal extracts at the highest concentration. Based on the color of the solution and mass spectrum data from the fluorescent extract, we also isolated a compound consistent with pheophytin α with peak absorption maximums around 410 and 665 nm and fluorescence around 670 nm [44,65]. We avoided fluorescence interferences that may have provided false-positives (indicative of PAINS-like compounds) by thoroughly understanding the fluorescent properties of the *K. brevis* extract and designed subsequent fluorescent-based cellular assays accordingly. 

Previous work by others indicated extracts of various algae species impact the cellular responses and regulate inflammatory states making marine algae an important source for novel natural therapies [17,18,19,20,21], yet their direct interaction with macrophages, the main initiators of the immune response and inflammation, remained unknown. The only known study that examined fluorescent natural product accumulation in target cells involved human lymphocytes which play an entirely different role in the immune response [66]. To understand how an immune response is initiated, macrophage activity must be robustly characterized. Both phagocytic RAW 264.7 macrophages and non-phagocytic Vero cells accumulated fluorescent material inside the cells and continued to accumulate as early as 30 min post incubation and peaked at 24 h through a 48 h time course. We showed fluorescent extract accumulation was dynamin-independent in the Vero kidney and RAW 264.7 macrophages. 

Blockade of acidified endosomes by EGA only inhibited uptake of the extract in the non-phagocytic kidney Vero cells. Together, these data suggest the *K. brevis* extract accumulates in phagocytic cells and non-phagocytic cells through different mechanisms. Notably, EGA treatment increased uptake of the extract in RAW 264.7 cells and Dynasore treatment increased uptake of the extract in Vero cells. It is not surprising that immune phagocytic cells such as RAW 264.7 cells would interact with the *K. brevis* extract through a different mechanism than non-phagocytic kidney Vero cells; however, the precise mechanism of the increase in accumulation due to these inhibitors is unclear to date. Researchers have demonstrated increased phagocytosis in RAW 264.7 cells due to nutrient deprivation or specific treatments [67,68]; however, it has yet to be discovered why EGA may increase RAW 264.7 phagocytosis. Dynasore can have off-target dynamin-independent effects on cells, particularly in the disruption of lipid rafts and decreasing labile cholesterol [69,70], which could explain the increase in uptake in Vero cells. However, further studies are needed on the potential activation effects of these two inhibitors. Regardless, these results still support the finding that *K. brevis* extract accumulates in these two cell types via different mechanisms.

Previous studies indicate macrophages phagocytose foreign components within a couple of minutes, the timing of which is largely dependent on target shape, structure/charge, and size [71]. These data are consistent with other studies, which have found that macrophages scavenge natural products [47,72]. Phagosomes undergo acidification prior to fusing with the cell’s lysosome, ultimately destroying the foreign particle [73]. It is probable that any chlorophyll α ingested by macrophages is acidified and pheophytin α is the main contributor to fluorescence within the macrophages. Furthermore, we observed a down-regulation of CD206 in extract-stimulated macrophages, further indicating extract is likely phagocytosed by the RAW 264.7 macrophages as opposed to using dynamin-dependent or endosomal trafficking mechanisms.

Algal extracts can not only stimulate a variety of cells, but cells can also accumulate extract components within the cells leading to an increased cellular fluorescence through distinct pathways for entry. The mechanism by which cells accumulate fluorescence can vary dramatically and influence cellular responses. Immune cells such as macrophages are designed to phagocytose potentially harmful compounds, including those of pathogenic and endogenous/inflammatory origin, whereas non-phagocytic cells such as Vero cells have the capacity to engulf algae extracts through non-phagocytic cellular mechanisms. Subsequent studies by our group will focus on elucidating the intracellular mechanisms responsible for internalization and downstream activation in macrophages. 

## 4. Materials and Methods

### 4.1. Algal Extracts

*Karenia brevis* cultures were grown and partitioned using 1 part ethyl acetate (Fisher Chemical, Fair Lawn, NJ, USA) per 5 parts *K. brevis* culture, followed by homogenization. The ethyl acetate partition was filtered, and the filtrate was partitioned using 90:10 methanol: petroleum ether (both Honeywell, Muskegon, MI, USA) liquid-liquid extraction. The petroleum ether fraction was then extracted using 90:10 acetonitrile (Fisher Chemical, Fair Lawn, NJ, USA):H_2_O. The insoluble non-polar residual was solubilized in hexane (Fisher Chemical, Fair Lawn, NJ, USA) and separated using a 24 g Silica RediSep column on a Combiflash Rf+ (Teledyne ISCO, Lincoln, NE, USA). A binary gradient of hexane (A) and acetone (B) with a flow rate of 35 mL/min was executed: 100% A for the first 3 min, linear change to 65.6% A and 34.4% B from 3 to 6.6 min, held from 6.6 to 9 min, linear change to 0% A and 100% B from 9 to 15.8 min, held until 25.5 min, and returned to 100% A from 25.5 to 28 min. Fractions were collected by 215 nm UV peaks and dried to completeness on a speedvac (Thermo Fisher Scientific, Waltham, MA, USA) for bioassay screening. Each fraction was resolubilized in ethanol for cell treatment. The fraction with the highest fluorescence response when added to the macrophage cell line was designated as the *K. brevis* extract and used for further testing. 

To identify the most abundant compound of the *K. brevis* extract, it was further purified using HPLC-UV (Agilent 1260 Infinity II) with a Thermo Scientific Hypersil MOS-2 C8 column (4.6 × 100 mm, 3 μm). A binary gradient with a flow rate of 1.0 mL/min was used for LC separation. Mobile phase A (MPA) consisted of 100% methanol and mobile phase B (MPB) consisted of 0.15 M ammonium acetate in 70:30 methanol:H_2_O. The elution gradient began at 25% MPA and 75% MPB and linearly changed to 50% MPA and 50% MPB by 1 min. From 1 to 15 min, the percentage of MPA linearly increased to 100% and was held at 100% until 18 min. From 18 to 19 min, MPA returned to 25% and MPB returned to 75% and was held at this ratio for 1 min. Compounds were detected at 230, 280, 440, 480, 650, 667, and 710 nm. The peak with the greatest area was collected with the UV-Vis lamp turned off to prevent any photo bleaching of the naturally occurring fluorescence. The molecular mass of the compound collected was determined via mass spectrometry (SCIEX QTRAP 4000) with an ESI source set in positive mode. 

### 4.2. Cell Culture

The murine macrophage cell line, RAW 264.7 (ATCC^®^ TIB-71, Manassas, VA, USA), was cultured in RPMI 1640 media (Corning, Manassas, VA, USA) with 10% heat inactivated fetal bovine serum (Avantor Seradigm, Randor, PA, USA), Antibiotic-Antimycotic (Gibco, Carlsbad, CA, USA), and 4.5 g/L D-glucose (Sigma-Aldrich, Saint Louis, MO, USA). 

African green monkey kidney cells, Vero (ATCC^®^ CCL-81, Manassas, VA, USA), were used as a non-phagocytic control. Cells were cultured in DMEM (Gibco, Carlsbad, CA, USA) with 10% heat inactivated fetal bovine serum (Avantor Seradigm, Randor, PA, USA), and Antibiotic-Antimycotic (Gibco, Carlsbad, CA, USA).

### 4.3. Flow Cytometry Analysis

RAW 264.7 cells were harvested by incubating in 1000 µL of iced Phosphate-Buffered Saline (PBS, Sigma Aldrich, St. Louis, MO, USA) for 20 min on ice. Non-specific Fc receptors were blocked with 75 µL goat serum (Gibco, Carlsbad, CA, USA) for 15 min and then stained with 100 µL fluorescent antibodies at 2 µg/mL. After 2 h incubation, cells were washed and resuspended in 400 µL of PBS and analyzed on BD FACSCelesta flow cytometer (BD Biosciences, San Jose, CA, USA). Cells were gated to exclude necrotic cells and aggregates as previously described [74]. Fluorescent antibody panels were designed as stated in the results. Antibodies included: (rat) anti-mannose receptor CD206 conjugated to Alexa Fluor 488 (R&D Systems, Minneapolis, MN, USA), (hampster) anti-mouse CD80 conjugated to PE-CF594 (BD Biosciences, San Jose, CA, USA), and (rat) anti-mouse IL4Rα conjugated to BV421 (BD Biosciences, San Jose, CA, USA). For screening of all flow cytometry filters, cells were harvested, rinsed twice with 1 mL iced PBS, and suspended with 0.5 mL of iced PBS and ran immediately on flow cytometer with all laser lines and filters monitoring. Vero cells were harvested using 0.25% Trypsin EDTA solution (Gibco, Carlsbad, CA, USA).

### 4.4. Spectral Analysis

Extracts were dissolved in ethanol (Fisher Chemical, Fair Lawn, NJ, USA) at concentrations ranging 500 ng/mL to 1 mg/mL for spectral analysis on FlexStation 3 (Molecular Devices, San Jose, CA, USA) using the SoftMax Pro 5.2 software (Molecular Devices, San Jose, CA, USA). Peak absorbance values were used as excitation values for fluorescence spectral determination, as were corresponding excitation wavelengths for the flow cytometer lasers.

### 4.5. Fluorescence Microscopy

Cells were plated in 96-well plates (Greiner Bio-One, Monroe, NC, USA) and treated with 500 ng/mL algal extracts. After 24 h, cells were treated with 100 ng/mL Hoechst nuclear stain (Invitrogen, Carlsbad, CA, USA) for one hour, washed, then imaged using Image Xpress PICO (Molecular Devices, San Jose, CA, USA) at 20× using DAPI and Cy5 filters.

### 4.6. Statistical Analysis

Differences in average fluorescence intensity for different treatments were compared using a one-way ANOVA with post hoc analysis on GraphPad Prism 7.05 software (San Diego, CA, USA). In all experiments, results are presented as the mean +/− standard deviation and were considered statistically significant if a *p*-value of less than 0.05 was obtained.

## Data Availability

The data presented in this study are available on request from the corresponding author.

## Supplementary Materials

The following supporting information can be downloaded at: https://www.mdpi.com/article/10.3390/md22010004/s1, Figure S1: Mass spectrum of isolated compound from *K. brevis* algae extract analyzed in positive mode from 850–925 Da indicates the presence of pheophytin α. Figure S2: Fluorescence accumulation in RAW 264.7 cells and Vero cells over a 30 min to 48 h time course as analyzed by the red laser line Alexa Fluor 647 filter. Table S1. Relative fluorescence change over untreated control cells with all flow cytometry filters.

## References

[1] Poli M.A., Mende T.J., Baden D.G. (1986). Brevetoxins, Unique Activators of Voltage-Sensitive Sodium Channels, Bind to Specific Sites in Rat Brain Synaptosomes. Mol. Pharmacol..

[2] Gannon D., Berens McCabe E., Camilleri S., Gannon J., Brueggen M., Barleycorn A., Palubok V., Kirkpatrick G., Wells R. (2012). Effects of *Karenia brevis* Harmful Algal Blooms on Nearshore Fish Communities in Southwest Florida. Mar. Ecol. Prog. Ser..

[3] Fleming L.E., Kirkpatrick B., Backer L.C., Walsh C.J., Nierenberg K., Clark J., Reich A., Hollenbeck J., Benson J., Cheng Y.S. (2011). Review of Florida Red Tide and Human Health Effects. Harmful Algae.

[4] Tilney C.L., Shankar S., Hubbard K.A., Corcoran A.A. (2019). Is *Karenia brevis* Really a Low-Light-Adapted Species?. Harmful Algae.

[5] Mendoza W.G., Kang Y., Zika R.G. (2012). Resolving DOM Fluorescence Fractions during a *Karenia brevis* Bloom Patch on the Southwest Florida Shelf. Cont. Shelf Res..

[6] Harris R.J., Arrington D.A., Porter D., Lovko V. (2020). Documenting the Duration and Chlorophyll Pigments of an Allochthonous *Karenia brevis* Bloom in the Loxahatchee River Estuary (LRE), Florida. Harmful Algae.

[7] Colman J.R., Ramsdell J.S. (2003). The Type B Brevetoxin (PbTx-3) Adversely Affects Development, Cardiovascular Function, and Survival in Medaka (*Oryzias latipes*) Embryos. Environ. Health Perspect..

[8] Abraham W.M., Bourdelais A.J., Sabater J.R., Ahmed A., Lee T.A., Serebriakov I., Baden D.G. (2005). Airway Responses to Aerosolized Brevetoxins in an Animal Model of Asthma. Am. J. Respir. Crit. Care Med..

[9] Pesek J.J., Matyska M.T., Hiltz T., McCall J. (2023). Application of a Cholesterol-Based Stationary Phase for the Analysis of Brevetoxins. J. Sep. Sci..

[10] Finol-Urdaneta R.K., Zhorov B.S., Baden D.G., Adams D.J. (2023). Brevetoxin versus Brevenal Modulation of Human Nav1 Channels. Mar. Drugs.

[11] Hort V., Abadie E., Arnich N., Dechraoui Bottein M.-Y., Amzil Z. (2021). Chemodiversity of Brevetoxins and Other Potentially Toxic Metabolites Produced by *Karenia* Spp. and Their Metabolic Products in Marine Organisms. Mar. Drugs.

[12] Keeler D.M., Grandal M.K., McCall J.R. (2019). Brevenal, a Marine Natural Product, Is Anti-Inflammatory and an Immunomodulator of Macrophage and Lung Epithelial Cells. Mar. Drugs.

[13] McCall J.R., Sausman K.T., Keeler D.M., Brown A.P., Turrise S.L. (2022). Immune Modulating Brevetoxins: Monocyte Cytotoxicity, Apoptosis, and Activation of M1/M2 Response Elements Is Dependent on Reactive Groups. Mar. Drugs.

[14] Jeglitsch G., Rein K., Baden D.G., Adams D.J. (1998). Brevetoxin-3 (PbTx-3) and Its Derivatives Modulate Single Tetrodotoxin-Sensitive Sodium Channels in Rat Sensory Neurons. J. Pharmacol. Exp. Ther..

[15] Chen W., Tuladhar A., Rolle S., Lai Y., del Rey F.R., Zavala C.E., Liu Y., Rein K.S. (2017). Brevetoxin-2, Is a Unique Inhibitor of the C-Terminal Redox Center of Mammalian Thioredoxin Reductase-1. Toxicol. Appl. Pharmacol..

[16] Tuladhar A., Hondal R.J., Colon R., Hernandez E.L., Rein K.S. (2019). Effectors of Thioredoxin Reductase: Brevetoxins and Manumycin-A. Comp. Biochem. Physiol. Toxicol. Pharmacol. CBP.

[17] Kang H., Lee C.H., Kim J.R., Kwon J.Y., Seo S.G., Han J.G., Kim B.G., Kim J.-E., Lee K.W. (2015). *Chlorella vulgaris* Attenuates Dermatophagoides Farinae-Induced Atopic Dermatitis-Like Symptoms in NC/Nga Mice. Int. J. Mol. Sci..

[18] Konishi F., Tanaka K., Himeno K., Taniguchi K., Nomoto K. (1985). Antitumor Effect Induced by a Hot Water Extract of *Chlorella vulgaris* (CE): Resistance to Meth-A Tumor Growth Mediated by CE-Induced Polymorphonuclear Leukocytes. Cancer Immunol. Immunother..

[19] Chovancikova M., Simek V. (2001). Effects of High-Fat and *Chlorella vulgaris* Feeding on Changes in Lipid Metabolism in Mice. Biologia.

[20] Cherng J.-Y., Shih M.-F. (2005). Potential Hypoglycemic Effects of Chlorella in Streptozotocin-Induced Diabetic Mice. Life Sci..

[21] Kaur M., Bhatia S., Gupta U., Decker E., Tak Y., Bali M., Gupta V.K., Dar R.A., Bala S. (2023). Microalgal Bioactive Metabolites as Promising Implements in Nutraceuticals and Pharmaceuticals: Inspiring Therapy for Health Benefits. Phytochem. Rev..

[22] Lee D., Nishizawa M., Shimizu Y., Saeki H. (2017). Anti-Inflammatory Effects of Dulse (*Palmaria palmata*) Resulting from the Simultaneous Water-Extraction of Phycobiliproteins and Chlorophyll a. Food Res. Int..

[23] Edwards B.S., Sklar L.A. (2015). Flow Cytometry: Impact on Early Drug Discovery. SLAS Discov..

[24] McKinnon K.M. (2018). Flow Cytometry: An Overview. Curr. Protoc. Immunol..

[25] Sun J., Kroeger J.L., Markowitz J. (2021). Introduction to Multiparametric Flow Cytometry and Analysis of High-Dimensional Data. Translational Bioinformatics for Therapeutic Development.

[26] Cossarizza A., Chang H.-D., Radbruch A., Acs A., Adam D., Adam-Klages S., Agace W.W., Aghaeepour N., Akdis M., Allez M. (2019). Guidelines for the Use of Flow Cytometry and Cell Sorting in Immunological Studies (Second Edition). Eur. J. Immunol..

[27] Baell J., Walters M.A. (2014). Chemistry: Chemical Con Artists Foil Drug Discovery. Nature.

[28] Capuzzi S.J., Muratov E.N., Tropsha A. (2017). Phantom PAINS: Problems with the Utility of Alerts for Pan-Assay INterference CompoundS. J. Chem. Inf. Model..

[29] van der Gronde T., Groot C.A.U., Pieters T. (2017). Addressing the Challenge of High-Priced Prescription Drugs in the Era of Precision Medicine: A Systematic Review of Drug Life Cycles, Therapeutic Drug Markets and Regulatory Frameworks. PLoS ONE.

[30] DiMasi J.A., Grabowski H.G., Hansen R.W. (2016). Innovation in the Pharmaceutical Industry: New Estimates of R&D Costs. J. Health Econ..

[31] Trindade I.C., Pound-Lana G., Pereira D.G.S., de Oliveira L.A.M., Andrade M.S., Vilela J.M.C., Postacchini B.B., Mosqueira V.C.F. (2018). Mechanisms of Interaction of Biodegradable Polyester Nanocapsules with Non-Phagocytic Cells. Eur. J. Pharm. Sci..

[32] Vercammen M., Scorza T., El Bouhdidi A., Van Beeck K., Carlier Y., Dubremetz J.F., Verschueren H., El Bouhdidi A., Van Beeck K. (1999). Opsonization of *Toxoplasma gondii* Tachyzoites with Nonspecific Immunoglobulins Promotes Their Phagocytosis by Macrophages and Inhibits Their Proliferation in Nonphagocytic Cells in Tissue Culture. Parasite Immunol..

[33] Atri C., Guerfali F.Z., Laouini D. (2018). Role of Human Macrophage Polarization in Inflammation during Infectious Diseases. Int. J. Mol. Sci..

[34] Funes S.C., Rios M., Escobar-Vera J., Kalergis A.M. (2018). Implications of Macrophage Polarization in Autoimmunity. Immunology.

[35] Murray P.J. (2017). Macrophage Polarization. Annu. Rev. Physiol..

[36] Gordon S., Martinez F.O. (2010). Alternative Activation of Macrophages: Mechanism and Functions. Immunity.

[37] Boutilier A.J., Elsawa S.F. (2021). Macrophage Polarization States in the Tumor Microenvironment. Int. J. Mol. Sci..

[38] Wang C., Ma C., Gong L., Guo Y., Fu K., Zhang Y., Zhou H., Li Y. (2021). Macrophage Polarization and Its Role in Liver Disease. Front. Immunol..

[39] Wang L., Zhang S., Wu H., Rong X., Guo J. (2019). M2b Macrophage Polarization and Its Roles in Diseases. J. Leukoc. Biol..

[40] Molecular Devices (2018). FlexStation 3 Multi-Mode Microplate Reader User Guide.

[41] Morançais M., Mouget J.-L., Dumay J., Levine I.A., Fleurence J. (2018). Chapter 7—Proteins and Pigments. Microalgae in Health and Disease Prevention.

[42] Mackinney G., Joslyn M.A. (1940). The Conversion of Chlorophyll to Pheophytin. J. Am. Chem. Soc..

[43] Petrovic S., Zvezdanović J., Anđelković T., Marković D. (2012). The Identification of Chlorophyll and Its Derivatives in the Pigment Mixtures: HPLC-Chromatography, Visible and Mass Spectroscopy Studies. Savrem. Technol..

[44] French C.S., Smith J.H.C., Virgin H.I., Airth R.L. (1956). Fluorescence-Spectrum Curves of Chlorophylls, Pheophytins, Phycoerythrins, Phycocyanins and Hypericin. Plant Physiol..

[45] Pathak C., Vaidya F.U., Waghela B.N., Jaiswara P.K., Gupta V.K., Kumar A., Rajendran B.K., Ranjan K. (2023). Insights of Endocytosis Signaling in Health and Disease. Int. J. Mol. Sci..

[46] França A., Aggarwal P., Barsov E.V., Kozlov S.V., Dobrovolskaia M.A., González-Fernández Á. (2011). Macrophage Scavenger Receptor A Mediates the Uptake of Gold Colloids by Macrophages In Vitro. Nanomedicine.

[47] Aderem A., Underhill D.M. (1999). Mechanisms of Phagocytosis in Macrophages. Annu. Rev. Immunol..

[48] Yue H., Wei W., Yue Z., Lv P., Wang L., Ma G., Su Z. (2010). Particle Size Affects the Cellular Response in Macrophages. Eur. J. Pharm. Sci..

[49] He C., Hu Y., Yin L., Tang C., Yin C. (2010). Effects of Particle Size and Surface Charge on Cellular Uptake and Biodistribution of Polymeric Nanoparticles. Biomaterials.

[50] Gillespie E.J., Ho C.-L.C., Balaji K., Clemens D.L., Deng G., Wang Y.E., Elsaesser H.J., Tamilselvam B., Gargi A., Dixon S.D. (2013). Selective Inhibitor of Endosomal Trafficking Pathways Exploited by Multiple Toxins and Viruses. Proc. Natl. Acad. Sci. USA.

[51] Macia E., Ehrlich M., Massol R., Boucrot E., Brunner C., Kirchhausen T. (2006). Dynasore, a Cell-Permeable Inhibitor of Dynamin. Dev. Cell.

[52] Shapouri-Moghaddam A., Mohammadian S., Vazini H., Taghadosi M., Esmaeili S.-A., Mardani F., Seifi B., Mohammadi A., Afshari J.T., Sahebkar A. (2018). Macrophage Plasticity, Polarization, and Function in Health and Disease. J. Cell. Physiol..

[53] Liu L., Guo H., Song A., Huang J., Zhang Y., Jin S., Li S., Zhang L., Yang C., Yang P. (2020). Progranulin Inhibits LPS-Induced Macrophage M1 Polarization via NF-κB and MAPK Pathways. BMC Immunol..

[54] Aki T., Funakoshi T., Noritake K., Unuma K., Uemura K. (2020). Extracellular Glucose Is Crucially Involved in the Fate Decision of LPS-Stimulated RAW264.7 Murine Macrophage Cells. Sci. Rep..

[55] Wen Z.-S., Xiang X.-W., Jin H.-X., Guo X.-Y., Liu L.-J., Huang Y.-N., OuYang X.-K., Qu Y.-L. (2016). Composition and Anti-Inflammatory Effect of Polysaccharides from Sargassum Horneri in RAW264.7 Macrophages. Int. J. Biol. Macromol..

[56] Yim M.-J., Lee J.M., Choi G., Lee D.-S., Park W.S., Jung W.-K., Park S., Seo S.-K., Park J., Choi I.-W. (2018). Anti-Inflammatory Potential of *Carpomitra costata* Ethanolic Extracts via Inhibition of NF-κB and AP-1 Activation in LPS-Stimulated RAW264.7 Macrophages. Evid.-Based Complement. Altern. Med..

[57] Porta C., Riboldi E., Ippolito A., Sica A. (2015). Molecular and Epigenetic Basis of Macrophage Polarized Activation. Semin. Immunol..

[58] Wang N., Liang H., Zen K. (2014). Molecular Mechanisms That Influence the Macrophage M1–M2 Polarization Balance. Front. Immunol..

[59] Mosser D.M., Edwards J.P. (2008). Exploring the Full Spectrum of Macrophage Activation. Nat. Rev. Immunol..

[60] Lee S.J., Evers S., Roeder D., Parlow A.F., Risteli J., Risteli L., Lee Y.C., Feizi T., Langen H., Nussenzweig M.C. (2002). Mannose Receptor-Mediated Regulation of Serum Glycoprotein Homeostasis. Science.

[61] Gazi U., Martinez-Pomares L. (2009). Influence of the Mannose Receptor in Host Immune Responses. Immunobiology.

[62] Liu H.-F., Zhang H.-J., Hu Q.-X., Liu X.-Y., Wang Z.-Q., Fan J.-Y., Zhan M., Chen F.-L. (2012). Altered Polarization, Morphology, and Impaired Innate Immunity Germane to Resident Peritoneal Macrophages in Mice with Long-Term Type 2 Diabetes. J. Biomed. Biotechnol..

[63] Sica A., Mantovani A. (2012). Macrophage Plasticity and Polarization: In Vivo Veritas. J. Clin. Investig..

[64] Daniel B., Nagy G., Czimmerer Z., Horvath A., Hammers D.W., Cuaranta-Monroy I., Poliska S., Tzerpos P., Kolostyak Z., Hays T.T. (2018). The Nuclear Receptor PPARγ Controls Progressive Macrophage Polarization as a Ligand-Insensitive Epigenomic Ratchet of Transcriptional Memory. Immunity.

[65] BD Biosciences (2020). BD FACSCelestaTM Flow Cytometer User’s Guide.

[66] Ottoni M.H.F., dos Santos M.G., de Almeida V.G., de Costa L.A., Meireles A.B., de Avelar-Freitas B.A., dos Santos J.A.T., de Fátima Pereira W., Brito-Melo G.E.A. (2019). Background Autofluorescence Induced by Plant Extracts in Human Lymphocytes: A Flow Cytometric Analysis of a Critical Bias. J. Immunol. Methods.

[67] Wu S., Romero-Ramírez L., Mey J. (2021). Retinoic Acid Increases Phagocytosis of Myelin by Macrophages. J. Cell. Physiol..

[68] Martinet W., Schrijvers D.M., Timmermans J.-P., Herman A.G., De Meyer G.R.Y. (2009). Phagocytosis of Bacteria Is Enhanced in Macrophages Undergoing Nutrient Deprivation. FEBS J..

[69] Clemente L.P., Rabenau M., Tang S., Stanka J., Cors E., Stroh J., Culmsee C., von Karstedt S. (2020). Dynasore Blocks Ferroptosis through Combined Modulation of Iron Uptake and Inhibition of Mitochondrial Respiration. Cells.

[70] Preta G., Cronin J.G., Sheldon I.M. (2015). Dynasore—Not Just a Dynamin Inhibitor. Cell Commun. Signal..

[71] Paul D., Achouri S., Yoon Y.-Z., Herre J., Bryant C.E., Cicuta P. (2013). Phagocytosis Dynamics Depends on Target Shape. Biophys. J..

[72] Greaves D.R., Gordon S. (2009). The Macrophage Scavenger Receptor at 30 Years of Age: Current Knowledge and Future Challenges. J. Lipid Res..

[73] McNeil P.L., Tanasugarn L., Meigs J.B., Taylor D.L. (1983). Acidification of Phagosomes Is Initiated before Lysosomal Enzyme Activity Is Detected. J. Cell Biol..

[74] McCall J.R., Sausman K.T. (2021). Systematic Approach in Macrophage Polarization Experiments: Maintaining Integrity and Reproducibility Using Flow Cytometry and Sample Preparation. J. Immunol. Methods.

