# Supplementary material for: Karenia brevis Extract Induces Cellular Entry through Distinct Mechanisms in Phagocytic RAW 264.7 Macrophages versus Non-Phagocytic Vero Cells
Source: Mar Drugs. 2023 Dec 19;22(1):4. doi: 10.3390/md22010004 (PMC10820030; doi:10.3390/md22010004)
Supplement: Supplementary file 1 [file marinedrugs-22-00004-s001.zip › marinedrugs-2771377-supplementary.pdf]

Supplementary Materials

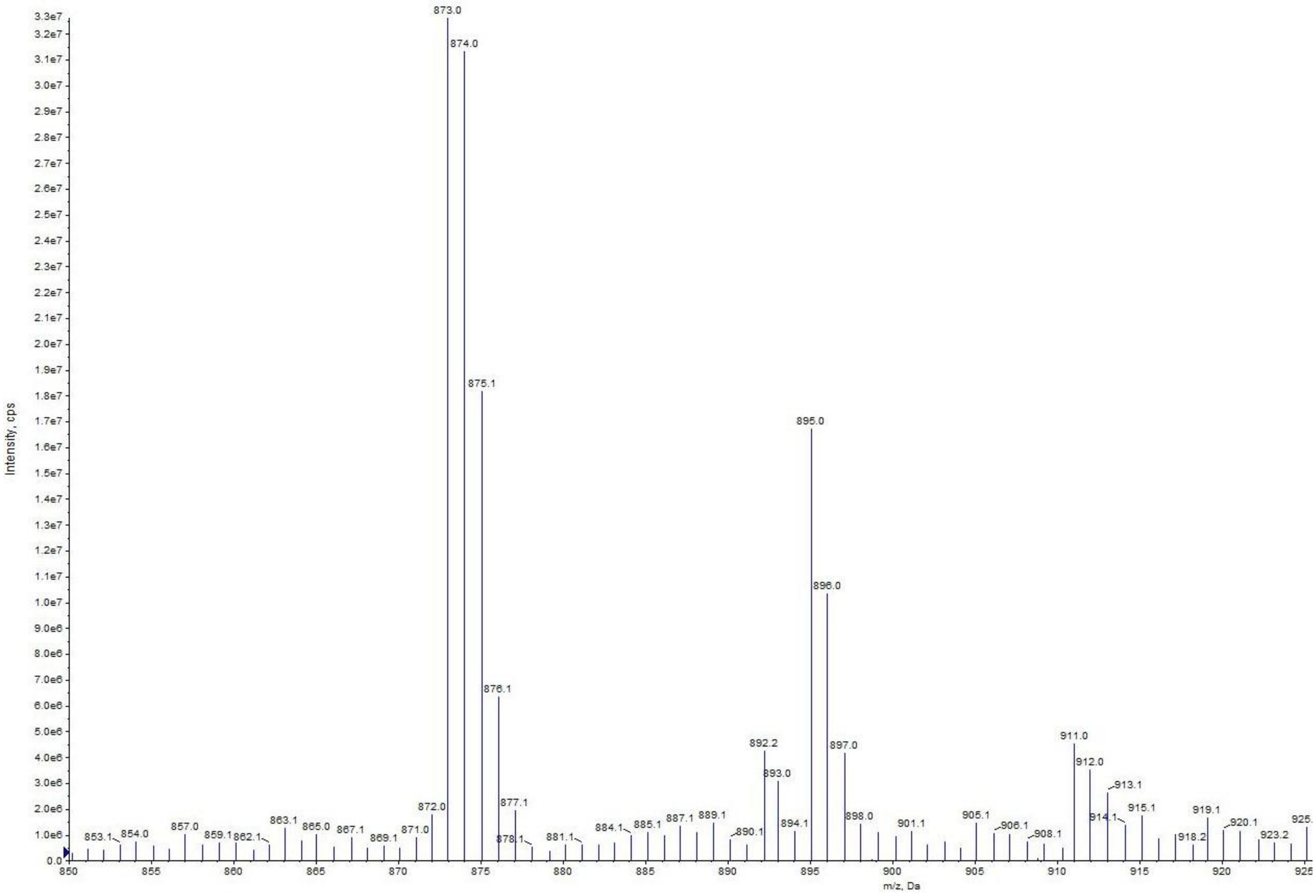

Figure S1. Mass spectrum of isolated compound from *K. brevis* algae extract analyzed in positive mode from 850 – 925 Da indicates the presence of pheophytin α.

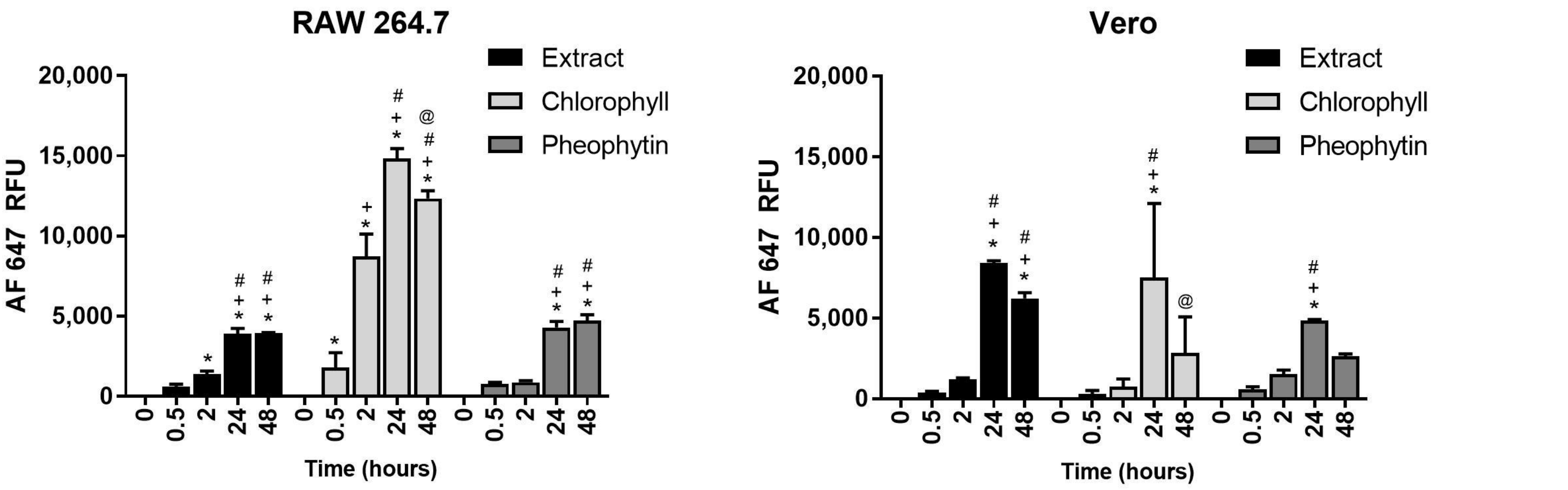

Figure S2. Fluorescence accumulation in RAW 264.7 cells and Vero cells over a 30 minute to 48 hour time course as analyzed by the red laser line Alexa Fluor 647 filter. Symbols indicate a statistically significant difference from no treatment (*), 0.5 hours (+), 2 hours (#), and 24 hours (@) with $p < 0.05$.

Table S1. Relative fluorescence change over untreated control cells with all flow cytometry filters. *K. Brevis* extract at 500 ng/mL on RAW 264.7 cells for 24 hours. Half of the channels were affected significantly by autofluorescence as determined by two-way ANOVA, * indicates a statistically significant difference from vehicle control (VC) with $p < 0.05$, (n=3).

Filter	Excitation Laser (nm)	Emission max (nm)	% Δ over control
BV421	Violet 405	421	-17%
BV510		510	-14%
BV605		602	-12%
BV650*		650	7,641%
BV786*		786	2,160%
BB515	Blue 488	515	-12%
PE		578	-17%
PE-CF594		612	-16%
PerCP*		678	1,260%
AF647*	Red 640	665	3,038%
APC*		660	1,094%
APC-C7*		785	589%
